# 7-Meth­oxy-2-phenyl­chroman-4-one

**DOI:** 10.1107/S1600536813001451

**Published:** 2013-01-23

**Authors:** Agata Piaskowska, Maciej Hodorowicz, Wojciech Nitek

**Affiliations:** a Faculty of Chemistry, Jagiellonian University, Ingardena 3, 30-060 Krakow, Poland

## Abstract

In the title compound, C_16_H_14_O_3_, the ring O atom and the two adjacent non-fused C atoms, as well as the attached phenyl ring, exhibit static disorder [occupancy ratio 0.559 (12):0.441 (12)]. The crystal packing features π–π [centroid–centroid distance = 3.912 (1) Å] and C—H⋯π inter­actions.

## Related literature

For aromatase inhibition of flavanones, see: Hong & Chen (2006 ▶). For the properties of 7-meth­oxy­flavanone, see: Pouget *et al.* (2001 ▶); Le Bail *et al.* (1998 ▶); Kostrzewa-Susłow *et al.* (2010 ▶). For classification of *X*—H⋯π inter­actions, see: Malone *et al.* (1997 ▶).
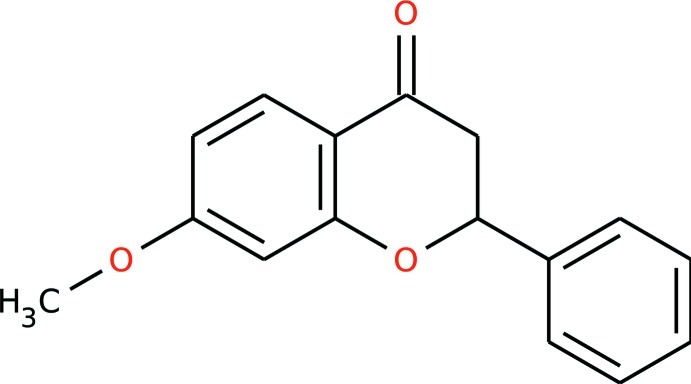


## Experimental

### 

#### Crystal data

C_16_H_14_O_3_
*M* *_r_* = 254.27
Monoclinic, 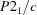

*a* = 8.5600 (3) Å
*b* = 6.6320 (2) Å
*c* = 23.4130 (7) Å
β = 90.742 (2)°
*V* = 1329.04 (7) Å^3^
*Z* = 4
Mo *K*α radiation
μ = 0.09 mm^−1^
*T* = 293 K
0.55 × 0.16 × 0.10 mm

#### Data collection

Nonius KappaCCD diffractometer
Absorption correction: multi-scan (*DENZO-SMN*; Otwinowski & Minor, 1997 ▶) *T*_min_ = 0.954, *T*_max_ = 0.991
15170 measured reflections
2710 independent reflections
1765 reflections with *I* > 2σ(*I*)
*R*_int_ = 0.072

#### Refinement

*R*[*F* ^2^ > 2σ(*F* ^2^)] = 0.086
*wR*(*F* ^2^) = 0.280
*S* = 1.18
2710 reflections
165 parameters
122 restraints
H-atom parameters constrained
Δρ_max_ = 0.65 e Å^−3^
Δρ_min_ = −0.35 e Å^−3^

### 

Data collection: *COLLECT* (Nonius, 1998 ▶); cell refinement: *SCALEPACK* (Otwinowski & Minor, 1997 ▶); data reduction: *DENZO* (Otwinowski & Minor, 1997 ▶) and *SCALEPACK*; program(s) used to solve structure: *SIR92* (Altomare *et al.*, 1994 ▶); program(s) used to refine structure: *SHELXL97* (Sheldrick, 2008 ▶); molecular graphics: *ORTEP-3* (Farrugia, 2012 ▶); software used to prepare material for publication: *publCIF* (Westrip, 2010 ▶).

## Supplementary Material

Crystal structure: contains datablock(s) I, global. DOI: 10.1107/S1600536813001451/kj2213sup1.cif

Structure factors: contains datablock(s) I. DOI: 10.1107/S1600536813001451/kj2213Isup2.hkl

Supplementary material file. DOI: 10.1107/S1600536813001451/kj2213Isup3.cml

Additional supplementary materials:  crystallographic information; 3D view; checkCIF report

## Figures and Tables

**Table 1:** Hydrogen-bond geometry (Å, °) *Cg*3, *Cg*4 and *Cg*5 are the centroids of the C5–C10, C11*A*–C16*A* and C11*B*–C16*B* rings, respectively.

*D*—H⋯*A*	*D*—H	H⋯*A*	*D*⋯*A*	*D*—H⋯*A*
C13*A*—H13*A*⋯*Cg*4^i^	0.93	2.80	3.598 (11)	144
C13*A*—H13*A*⋯*Cg*5^i^	0.93	2.71	3.515 (11)	146
C13*B*—H13*B*⋯*Cg*4^i^	0.93	2.82	3.695 (12)	158
C13*B*—H13*B*⋯*Cg*5^i^	0.93	2.76	3.639 (13)	157
C19—H19*B*⋯*Cg*4^ii^	0.96	2.72	3.619 (7)	156
C19—H19*B*⋯*Cg*5^ii^	0.96	2.76	3.660 (7)	157
C15*B*—H15*B*⋯*Cg*3^iii^	0.93	2.65	3.497 (14)	151

Symmetry codes: (i) 
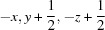
; (ii) 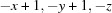; (iii) 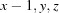.

**Fig. 1.**
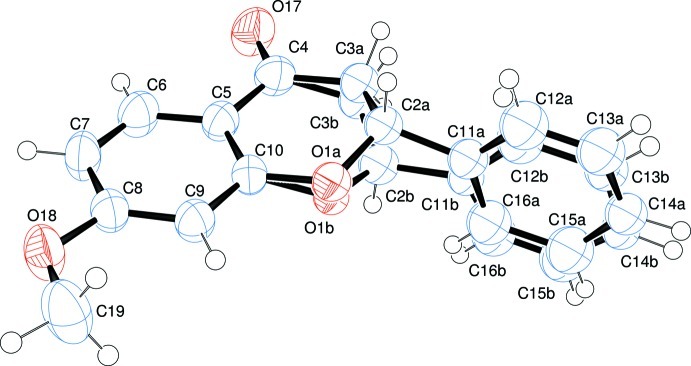
ORTEP-3 (Farrugia, 2012) drawing of the title compound with labels. Displacement ellipsoids of non-H atoms drawn at 30% probabilty level.

**Fig. 2.**
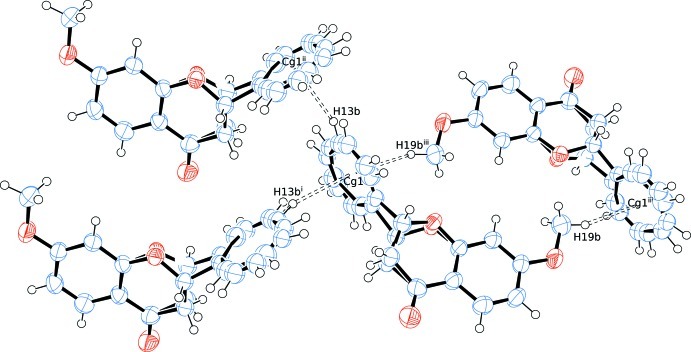
C—H···π and interactions in the crystal packing. Thermal ellipsoids are drawn at the 30% probability level. [Symmetry codes: (i) -x, y-&frac12;, &frac12;-z, (ii) -x, y+&frac12;, &frac12;-z, (iii) 1 - x, 1 - y, -z.]

**Fig. 3.**
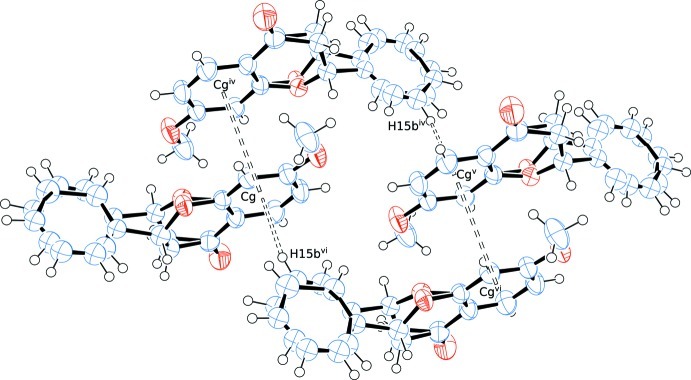
π–π interactions in the crystal packing. Thermal ellipsoids are drawn at the 30% probability level. [Symmetry codes: (iv) 1 - x, -y, -z, (v) 2 - x, -y, -z, (vi) x + 1, y, z.]

## supplementary crystallographic information

### Comment

Flavanones are of interest because of their anticancer effect as the aromatase inhibitors. By competing with androgens for binding with aromatase these compounds prevent the hydroxylation of C18 androgens to aromatic C19 estrogenic steroids (Hong & Chen, 2006). This suppresses the overexpression of aromatase in breast cancer (Pouget *et al.*, 2001).

The heterocyclic ring contains O1, C2 and C3 atoms exhibiting static disorder. This disorder is propagated into the attached phenyl. The dihedral angle between the C5—C10 aromatic ring plane and the phenyl ring plane is 88.6 (1)° for the major disorder component (C11A—C16A) and 87.3 (1)° for the minor component (C11B—C16B). The structure is stabilized by π–π and C—H···π interactions (Table 1). The C5—C10 ring displays a π–π interaction with the C5^i^—C10^i^ ring (Fig. 2 b) with a perpendicular distance of 3.543 (1) Å, a centroid-to-centroid distance of 3.912 (1) Å and a slippage of 1.658 Å [symmetry code: (i) 1 - *x*, -*y*, -*z*]. There are three types of C—H···π interactions: C13A—H13A···*Cg*4^i^ (C13B—13B··· *Cg*4^i^ in the minor disorder component), C19—H19B··· *Cg*4^ii^ (Fig. 2a) and C15B—H15B···*Cg*3^iii^ (Fig. 2 a) [symmetry codes: (i) -*x*, *y*+1/2, 1/2-*z*, (ii) 1 - *x*, 1 - *y*, -*z*, (iii) *x* - 1, *y*, *z*]. The first interaction falls into type III *X*—H···pi interactions while the rest can be classified as type I according to Malone and coworkers (Malone *et al.* (1997)).

### Experimental

The title compound was purchased from Sigma-Aldrich and used without further purification. Single crystals were obtained by slow evaporation of MeOH solution.

### Refinement

All hydrogen atom positions were observed in difference Fourier map. Nevertheless, in the refinement procedure the hydrogen atoms were positioned geometrically and refined using a riding model (including free rotation about the C—C bond for CH_3_groups), with C—H = 0.93—0.96 Å (C—H = 0.97 Å for CH_2_ groups, 0.96 Å for CH_3_ groups, and 0.93 Å for aromatic CH) and with *U*_iso_(H) = 1.5*U*_eq_(C) for methyl groups and *U*_iso_(H) = 1.2*U*_eq_(C) for all other H atoms. Disordered non-H atoms were refined with isotropic displacement parameters.

### Crystal data

**Table:**

C_16_H_14_O_3_	*F*(000) = 536
*M**_r_* = 254.27	*D*_x_ = 1.271 Mg m^−^^3^
Monoclinic, *P*2_1_/*c*	Mo *K*α radiation, λ = 0.71073 Å
Hall symbol: -P 2ybc	Cell parameters from 13054 reflections
*a* = 8.5600 (3) Å	θ = 0.4–26.4°
*b* = 6.6320 (2) Å	µ = 0.09 mm^−^^1^
*c* = 23.4130 (7) Å	*T* = 293 K
β = 90.742 (2)°	Prism, colourless
*V* = 1329.04 (7) Å^3^	0.55 × 0.16 × 0.10 mm
*Z* = 4	

### Data collection

**Table:**

Nonius KappaCCD diffractometer	2710 independent reflections
Radiation source: fine-focus sealed tube	1765 reflections with *I* > 2σ(*I*)
Graphite monochromator	*R*_int_ = 0.072
Detector resolution: 9 pixels mm^-1^	θ_max_ = 26.4°, θ_min_ = 2.9°
CCD scans	*h* = −8→10
Absorption correction: multi-scan (*DENZO-SMN*; Otwinowski & Minor, 1997)	*k* = −8→8
*T*_min_ = 0.954, *T*_max_ = 0.991	*l* = −29→29
15170 measured reflections	

### Refinement

**Table:**

Refinement on *F*^2^	Primary atom site location: structure-invariant direct methods
Least-squares matrix: full	Secondary atom site location: difference Fourier map
*R*[*F*^2^ > 2σ(*F*^2^)] = 0.086	Hydrogen site location: inferred from neighbouring sites
*wR*(*F*^2^) = 0.280	H-atom parameters constrained
*S* = 1.18	*w* = 1/[σ^2^(*F*_o_^2^) + (0.1433*P*)^2^ + 0.4478*P*] where *P* = (*F*_o_^2^ + 2*F*_c_^2^)/3
2710 reflections	(Δ/σ)_max_ < 0.001
165 parameters	Δρ_max_ = 0.65 e Å^−^^3^
122 restraints	Δρ_min_ = −0.35 e Å^−^^3^

### Special details

**Table:**

Geometry. All e.s.d.'s (except the e.s.d. in the dihedral angle between two l.s. planes) are estimated using the full covariance matrix. The cell e.s.d.'s are taken into account individually in the estimation of e.s.d.'s in distances, angles and torsion angles; correlations between e.s.d.'s in cell parameters are only used when they are defined by crystal symmetry. An approximate (isotropic) treatment of cell e.s.d.'s is used for estimating e.s.d.'s involving l.s. planes.
Refinement. Refinement of *F*^2^ against ALL reflections. The weighted *R*-factor *wR* and goodness of fit *S* are based on *F*^2^, conventional *R*-factors *R* are based on *F*, with *F* set to zero for negative *F*^2^. The threshold expression of *F*^2^ > σ(*F*^2^) is used only for calculating *R*-factors(gt) *etc*. and is not relevant to the choice of reflections for refinement. *R*-factors based on *F*^2^ are statistically about twice as large as those based on *F*, and *R*- factors based on ALL data will be even larger.

### Fractional atomic coordinates and isotropic or equivalent isotropic displacement parameters (Å^2^)

**Table:**

	*x*	*y*	*z*	*U*_iso_*/*U*_eq_	Occ. (<1)
O17	0.5477 (4)	−0.2944 (5)	0.18360 (14)	0.0728 (11)	
O18	0.8157 (4)	0.1840 (5)	−0.02843 (13)	0.0638 (9)	
C4	0.5036 (5)	−0.1399 (6)	0.15982 (17)	0.0534 (11)	
C5	0.5802 (5)	−0.0580 (6)	0.10887 (16)	0.0476 (10)	
C6	0.7077 (5)	−0.1537 (7)	0.08388 (19)	0.0592 (12)	
H6	0.7426	−0.2759	0.0988	0.071*	
C7	0.7821 (5)	−0.0726 (7)	0.03831 (18)	0.0577 (12)	
H7	0.8668	−0.1386	0.0224	0.069*	
C8	0.7298 (5)	0.1117 (6)	0.01556 (16)	0.0483 (10)	
C9	0.6034 (5)	0.2073 (6)	0.03813 (16)	0.0483 (10)	
H9	0.5684	0.3286	0.0227	0.058*	
C10	0.5271 (4)	0.1219 (6)	0.08444 (15)	0.0429 (9)	
C19	0.7766 (7)	0.3789 (8)	−0.0506 (2)	0.0777 (16)	
H19A	0.6695	0.3793	−0.0634	0.116*	
H19B	0.8431	0.4098	−0.0822	0.116*	
H19C	0.7908	0.4784	−0.0213	0.116*	
O1A	0.4127 (8)	0.2362 (10)	0.1081 (3)	0.043 (2)*	0.559 (12)
C2A	0.3587 (8)	0.1876 (10)	0.1623 (3)	0.043 (2)*	0.559 (12)
H2A	0.4289	0.2677	0.1867	0.052*	0.559 (12)
C3A	0.3801 (12)	−0.0050 (14)	0.1850 (4)	0.049 (3)*	0.559 (12)
H3A1	0.2810	−0.0754	0.1819	0.059*	0.559 (12)
H3A2	0.4039	0.0100	0.2254	0.059*	0.559 (12)
C11A	0.2053 (10)	0.2932 (13)	0.1710 (4)	0.047 (2)*	0.559 (12)
C12A	0.2038 (13)	0.4478 (18)	0.2065 (6)	0.065 (4)*	0.559 (12)
H12A	0.2961	0.4805	0.2258	0.078*	0.559 (12)
C13A	0.0813 (12)	0.5567 (15)	0.2160 (4)	0.062 (3)*	0.559 (12)
H13A	0.0901	0.6657	0.2408	0.074*	0.559 (12)
C14A	−0.0591 (11)	0.5178 (13)	0.1912 (4)	0.050 (2)*	0.559 (12)
H14A	−0.1453	0.5977	0.1992	0.060*	0.559 (12)
C15A	−0.0727 (12)	0.3545 (19)	0.1530 (5)	0.068 (4)*	0.559 (12)
H15A	−0.1678	0.3222	0.1356	0.082*	0.559 (12)
C16A	0.0671 (13)	0.2391 (16)	0.1419 (5)	0.061 (3)*	0.559 (12)
H16A	0.0655	0.1322	0.1162	0.073*	0.559 (12)
O1B	0.3856 (10)	0.2064 (12)	0.0994 (3)	0.036 (2)*	0.441 (12)
C2B	0.2931 (11)	0.1055 (15)	0.1386 (4)	0.050 (3)*	0.441 (12)
H2B	0.2276	0.0244	0.1128	0.060*	0.441 (12)
C3B	0.3513 (14)	−0.0439 (19)	0.1748 (5)	0.046 (3)*	0.441 (12)
H3B1	0.2734	−0.1497	0.1770	0.055*	0.441 (12)
H3B2	0.3624	0.0137	0.2127	0.055*	0.441 (12)
C11B	0.1728 (13)	0.2564 (14)	0.1603 (4)	0.040 (3)*	0.441 (12)
C12B	0.1843 (14)	0.424 (2)	0.1974 (7)	0.062 (5)*	0.441 (12)
H12B	0.2792	0.4617	0.2140	0.074*	0.441 (12)
C13B	0.0404 (14)	0.5362 (17)	0.2086 (5)	0.052 (3)*	0.441 (12)
H13B	0.0393	0.6463	0.2333	0.062*	0.441 (12)
C14B	−0.0916 (11)	0.4712 (16)	0.1813 (4)	0.044 (3)*	0.441 (12)
H14B	−0.1852	0.5388	0.1871	0.053*	0.441 (12)
C15B	−0.0895 (15)	0.313 (2)	0.1463 (7)	0.068 (5)*	0.441 (12)
H15B	−0.1818	0.2747	0.1280	0.082*	0.441 (12)
C16B	0.0329 (14)	0.213 (2)	0.1373 (6)	0.063 (5)*	0.441 (12)
H16B	0.0256	0.1026	0.1130	0.076*	0.441 (12)

### Atomic displacement parameters (Å^2^)

**Table:**

	*U*^11^	*U*^22^	*U*^33^	*U*^12^	*U*^13^	*U*^23^
O17	0.072 (2)	0.069 (2)	0.078 (2)	0.0210 (17)	0.0127 (17)	0.0313 (17)
O18	0.065 (2)	0.063 (2)	0.0643 (18)	0.0106 (15)	0.0277 (15)	0.0115 (14)
C4	0.054 (3)	0.053 (2)	0.053 (2)	0.004 (2)	0.0016 (19)	0.0095 (19)
C5	0.045 (2)	0.048 (2)	0.050 (2)	0.0077 (18)	0.0035 (17)	0.0043 (17)
C6	0.058 (3)	0.053 (2)	0.066 (3)	0.015 (2)	0.007 (2)	0.011 (2)
C7	0.052 (3)	0.059 (3)	0.063 (3)	0.015 (2)	0.017 (2)	0.003 (2)
C8	0.045 (2)	0.053 (2)	0.047 (2)	0.0039 (18)	0.0085 (17)	0.0032 (17)
C9	0.050 (2)	0.046 (2)	0.049 (2)	0.0081 (18)	0.0058 (18)	0.0056 (16)
C10	0.041 (2)	0.046 (2)	0.0426 (19)	0.0041 (16)	0.0048 (16)	−0.0013 (16)
C19	0.095 (4)	0.065 (3)	0.074 (3)	0.008 (3)	0.036 (3)	0.016 (2)

### Geometric parameters (Å, º)

**Table:**

O17—C4	1.224 (5)	C11A—C16A	1.404 (12)
O18—C8	1.361 (5)	C12A—C13A	1.295 (11)
O18—C19	1.432 (6)	C12A—H12A	0.9300
C4—C5	1.472 (6)	C13A—C14A	1.353 (11)
C4—C3B	1.496 (12)	C13A—H13A	0.9300
C4—C3A	1.510 (10)	C14A—C15A	1.409 (12)
C5—C10	1.397 (5)	C14A—H14A	0.9300
C5—C6	1.398 (6)	C15A—C16A	1.447 (12)
C6—C7	1.360 (6)	C15A—H15A	0.9300
C6—H6	0.9300	C16A—H16A	0.9300
C7—C8	1.404 (6)	O1B—C2B	1.392 (11)
C7—H7	0.9300	C2B—C3B	1.391 (14)
C8—C9	1.366 (5)	C2B—C11B	1.527 (13)
C9—C10	1.393 (5)	C2B—H2B	0.9800
C9—H9	0.9300	C3B—H3B1	0.9700
C10—O1A	1.362 (7)	C3B—H3B2	0.9700
C10—O1B	1.384 (8)	C11B—C16B	1.337 (13)
C19—H19A	0.9600	C11B—C12B	1.414 (13)
C19—H19B	0.9600	C12B—C13B	1.465 (13)
C19—H19C	0.9600	C12B—H12B	0.9300
O1A—C2A	1.394 (9)	C13B—C14B	1.362 (13)
C2A—C3A	1.395 (11)	C13B—H13B	0.9300
C2A—C11A	1.504 (10)	C14B—C15B	1.330 (13)
C2A—H2A	0.9800	C14B—H14B	0.9300
C3A—H3A1	0.9700	C15B—C16B	1.260 (13)
C3A—H3A2	0.9700	C15B—H15B	0.9300
C11A—C12A	1.321 (12)	C16B—H16B	0.9300
			
C8—O18—C19	117.8 (3)	C12A—C11A—C2A	117.5 (8)
O17—C4—C5	122.6 (4)	C16A—C11A—C2A	123.2 (8)
O17—C4—C3B	121.0 (5)	C13A—C12A—C11A	123.8 (10)
C5—C4—C3B	115.5 (5)	C13A—C12A—H12A	118.1
O17—C4—C3A	122.1 (5)	C11A—C12A—H12A	118.1
C5—C4—C3A	114.8 (5)	C12A—C13A—C14A	122.4 (9)
C3B—C4—C3A	16.3 (6)	C12A—C13A—H13A	118.8
C10—C5—C6	117.9 (4)	C14A—C13A—H13A	118.8
C10—C5—C4	120.1 (3)	C13A—C14A—C15A	119.0 (7)
C6—C5—C4	122.0 (4)	C13A—C14A—H14A	120.5
C7—C6—C5	121.6 (4)	C15A—C14A—H14A	120.5
C7—C6—H6	119.2	C14A—C15A—C16A	117.3 (8)
C5—C6—H6	119.2	C14A—C15A—H15A	121.4
C6—C7—C8	119.4 (4)	C16A—C15A—H15A	121.4
C6—C7—H7	120.3	C11A—C16A—C15A	118.1 (8)
C8—C7—H7	120.3	C11A—C16A—H16A	120.9
O18—C8—C9	124.6 (4)	C15A—C16A—H16A	120.9
O18—C8—C7	114.9 (3)	C10—O1B—C2B	118.7 (6)
C9—C8—C7	120.5 (4)	C3B—C2B—O1B	122.8 (9)
C8—C9—C10	119.6 (4)	C3B—C2B—C11B	120.2 (8)
C8—C9—H9	120.2	O1B—C2B—C11B	107.2 (7)
C10—C9—H9	120.2	C3B—C2B—H2B	100.5
O1A—C10—O1B	15.2 (4)	O1B—C2B—H2B	100.5
O1A—C10—C9	115.9 (4)	C11B—C2B—H2B	100.5
O1B—C10—C9	116.9 (4)	C2B—C3B—C4	117.9 (8)
O1A—C10—C5	122.8 (4)	C2B—C3B—H3B1	107.8
O1B—C10—C5	121.6 (4)	C4—C3B—H3B1	107.8
C9—C10—C5	120.9 (3)	C2B—C3B—H3B2	107.8
O18—C19—H19A	109.5	C4—C3B—H3B2	107.8
O18—C19—H19B	109.5	H3B1—C3B—H3B2	107.2
H19A—C19—H19B	109.5	C16B—C11B—C12B	118.0 (8)
O18—C19—H19C	109.5	C16B—C11B—C2B	109.3 (9)
H19A—C19—H19C	109.5	C12B—C11B—C2B	132.7 (10)
H19B—C19—H19C	109.5	C11B—C12B—C13B	117.3 (9)
C10—O1A—C2A	119.3 (5)	C11B—C12B—H12B	121.4
O1A—C2A—C3A	121.0 (6)	C13B—C12B—H12B	121.4
O1A—C2A—C11A	108.4 (6)	C14B—C13B—C12B	116.6 (9)
C3A—C2A—C11A	119.0 (7)	C14B—C13B—H13B	121.7
O1A—C2A—H2A	101.4	C12B—C13B—H13B	121.7
C3A—C2A—H2A	101.4	C15B—C14B—C13B	121.5 (9)
C11A—C2A—H2A	101.4	C15B—C14B—H14B	119.3
C2A—C3A—C4	118.9 (7)	C13B—C14B—H14B	119.3
C2A—C3A—H3A1	107.6	C16B—C15B—C14B	122.3 (11)
C4—C3A—H3A1	107.6	C16B—C15B—H15B	118.8
C2A—C3A—H3A2	107.6	C14B—C15B—H15B	118.8
C4—C3A—H3A2	107.6	C15B—C16B—C11B	124.3 (11)
H3A1—C3A—H3A2	107.0	C15B—C16B—H16B	117.9
C12A—C11A—C16A	119.3 (8)	C11B—C16B—H16B	117.9
			
O17—C4—C5—C10	−178.3 (4)	O1A—C2A—C11A—C12A	−107.5 (10)
C3B—C4—C5—C10	12.2 (8)	C3A—C2A—C11A—C12A	108.8 (11)
C3A—C4—C5—C10	−5.9 (7)	O1A—C2A—C11A—C16A	70.2 (10)
O17—C4—C5—C6	0.8 (7)	C3A—C2A—C11A—C16A	−73.4 (12)
C3B—C4—C5—C6	−168.7 (7)	C16A—C11A—C12A—C13A	−0.8 (19)
C3A—C4—C5—C6	173.3 (6)	C2A—C11A—C12A—C13A	177.1 (11)
C10—C5—C6—C7	2.1 (7)	C11A—C12A—C13A—C14A	2 (2)
C4—C5—C6—C7	−177.1 (4)	C12A—C13A—C14A—C15A	−0.8 (16)
C5—C6—C7—C8	−0.1 (7)	C13A—C14A—C15A—C16A	−1.0 (16)
C19—O18—C8—C9	3.7 (6)	C12A—C11A—C16A—C15A	−1.0 (16)
C19—O18—C8—C7	−175.1 (4)	C2A—C11A—C16A—C15A	−178.8 (9)
C6—C7—C8—O18	177.5 (4)	C14A—C15A—C16A—C11A	1.8 (16)
C6—C7—C8—C9	−1.3 (7)	C9—C10—O1B—C2B	169.1 (7)
O18—C8—C9—C10	−178.0 (4)	C5—C10—O1B—C2B	−1.6 (10)
C7—C8—C9—C10	0.7 (6)	C10—O1B—C2B—C3B	17.8 (14)
C8—C9—C10—O1A	173.5 (5)	C10—O1B—C2B—C11B	163.4 (7)
C8—C9—C10—O1B	−169.5 (5)	O1B—C2B—C3B—C4	−17.8 (16)
C8—C9—C10—C5	1.3 (6)	C11B—C2B—C3B—C4	−159.3 (9)
C6—C5—C10—O1A	−174.3 (5)	O17—C4—C3B—C2B	−167.1 (8)
C4—C5—C10—O1A	4.9 (7)	C5—C4—C3B—C2B	2.6 (13)
C6—C5—C10—O1B	167.7 (6)	C3B—C2B—C11B—C16B	−107.0 (13)
C4—C5—C10—O1B	−13.1 (7)	O1B—C2B—C11B—C16B	106.3 (11)
C6—C5—C10—C9	−2.7 (6)	C3B—C2B—C11B—C12B	74.0 (17)
C4—C5—C10—C9	176.5 (4)	O1B—C2B—C11B—C12B	−72.7 (15)
C9—C10—O1A—C2A	−163.7 (5)	C16B—C11B—C12B—C13B	1.1 (19)
C5—C10—O1A—C2A	8.3 (9)	C2B—C11B—C12B—C13B	−180.0 (10)
C10—O1A—C2A—C3A	−20.7 (11)	C11B—C12B—C13B—C14B	−1.4 (19)
C10—O1A—C2A—C11A	−163.5 (6)	C12B—C13B—C14B—C15B	0.4 (18)
O1A—C2A—C3A—C4	19.2 (13)	C13B—C14B—C15B—C16B	1 (2)
C11A—C2A—C3A—C4	158.2 (7)	C14B—C15B—C16B—C11B	−1 (3)
O17—C4—C3A—C2A	166.8 (7)	C12B—C11B—C16B—C15B	0 (2)
C5—C4—C3A—C2A	−5.7 (11)	C2B—C11B—C16B—C15B	−178.9 (15)

### Hydrogen-bond geometry (Å, º)

*Cg*3, *Cg*4 and *Cg*5 are the centroid of the C5–C10, C11A–C16A and C11B–C16B rings, respectively.

**Table:**

*D*—H···*A*	*D*—H	H···*A*	*D*···*A*	*D*—H···*A*
C13*A*—H13*A*···*Cg*4^i^	0.93	2.80	3.598 (11)	144
C13*A*—H13*A*···*Cg*5^i^	0.93	2.71	3.515 (11)	146
C13*B*—H13*B*···*Cg*4^i^	0.93	2.82	3.695 (12)	158
C13*B*—H13*B*···*Cg*5^i^	0.93	2.76	3.639 (13)	157
C19—H19*B*···*Cg*4^ii^	0.96	2.72	3.619 (7)	156
C19—H19*B*···*Cg*5^ii^	0.96	2.76	3.660 (7)	157
C15*B*—H15*B*···*Cg*3^iii^	0.93	2.65	3.497 (14)	151

Symmetry codes: (i) −*x*, *y*+1/2, −*z*+1/2; (ii) −*x*+1, −*y*+1, −*z*; (iii) *x*−1, *y*, *z*.
